# STI knowledge and risk behaviors among university students in northeastern Italy: the role of medical education and sexual experience

**DOI:** 10.3389/fpubh.2026.1797397

**Published:** 2026-04-30

**Authors:** Ilaria Tocco Tussardi, Lucia Cazzoletti, Isolde Martina Busch, Silvia Poli, Roberto Benoni, Michela Rimondini, Stefano Tardivo, Francesca Moretti

**Affiliations:** 1 Section of Hygiene, Department of Diagnostics and Public Health, University of Verona, Verona, Italy; 2 Section of Epidemiology and Medical Statistics, Department of Diagnostics and Public Health, University of Verona, Verona, Italy; 3 Department of Neurosciences, Biomedicine and Movement Sciences, University of Verona, Verona, Italy; 4 Department of Public Health and Infectious Diseases, University “La Sapienza”, Rome, Italy

**Keywords:** health education, health knowledge, Italy, medical students, risk assessment, sexual behavior, sexually transmitted infections, university students

## Abstract

**Introduction:**

Sexually transmitted infections (STIs) represent a major public health concern among young adults, particularly university students. Despite high educational attainment, this population often shows important gaps in STI-related knowledge and inconsistencies between knowledge and preventive behaviors. Understanding how objective and self-perceived STI knowledge varies across educational backgrounds and sexual risk profiles is essential for designing effective, targeted prevention strategies.

**Methods:**

We conducted a cross-sectional survey among all students enrolled at the University of Verona (Italy) during the 2020–2021 academic year. An ad-hoc, anonymous online questionnaire assessed sociodemographic characteristics, STI-related knowledge, information sources, sexual behaviors, and self-perceived competence. Sexual exposure risk was classified into four profiles (minimal, low, medium, high) based on sexual activity status, age at sexual debut, and number of partners in the past 12 months. Multivariable logistic regression models examined factors associated with four outcomes: awareness of specific STIs, correct knowledge of preventive measures, and self-perceived knowledge about STI acquisition and transmission.

**Results:**

A total of 2,187 students were included (mean age 21.9 years; 76.2% female). Although all participants had heard of STIs, only 7.6% were aware of all infections considered. Consistent condom use was reported by 38.9% of sexually active students and was lowest among those with multiple partners. Medical students showed substantially higher objective and self-perceived knowledge across all domains, including a markedly increased likelihood of recognizing all STIs. Higher sexual risk profiles were also associated with better STI awareness and higher self-perceived knowledge. Age was positively associated with objective knowledge but not with self-perceived competence. Sex and sexual orientation were not independently associated with any knowledge outcomes.

**Discussion:**

STI-related knowledge among university students is strongly shaped by educational pathway and sexual experience. While medical students and sexually active individuals demonstrate greater awareness, substantial knowledge gaps and a disconnection between knowledge and protective behaviors persist, particularly regarding condom use. These findings highlight the need for comprehensive, university-wide sexual health interventions that address both knowledge and behavioral determinants to effectively reduce STI risk among young adults.

## 1 Introduction

Sexually transmitted infections (STIs) remain a major global public health concern. These infections are transmitted primarily through sexual contact - vaginal, oral, and anal intercourse - as well as through intimate skin-to-skin contact, as seen with herpes simplex virus. A critical challenge in STI control is that many infections present with minimal or no symptoms, leading to delayed diagnosis and continued transmission.

The scale of the problem is substantial. According to the World Health Organization (WHO), more than one million new curable STI infections occur daily worldwide, caused by over 30 different bacteria, viruses, and parasites (1). Global estimates from 2020 documented hundreds of millions of new infections due to *Chlamydia trachomatis*, *Neisseria gonorrhoeae*, *Treponema pallidum*, and Trichomonas vaginalis (2). Despite advances in prevention and treatment, both curable and non-curable STIs continue to impose a substantial public health burden, with marked variation across geographical regions and population groups.

Adolescents and young adults aged 15–24 years face disproportionately high risks of STI acquisition. In the United States, surveillance data from the Centers for Disease Control and Prevention (CDC) indicate that nearly half of all newly diagnosed STIs occur in this age group (3). Similarly, European surveillance by the European Centre for Disease Prevention and Control (ECDC) shows increasing trends for several STIs, particularly chlamydia and gonorrhoea, with young adults accounting for a substantial proportion of cases across EU/EEA countries (4).

Italian surveillance data mirror these international patterns. The Istituto Superiore di Sanità (Italian National Institute of Health, ISS) coordinates STI surveillance through sentinel systems established in the early 1990s. Recent reports show an increase in STI cases compared with previous decades, with young people remaining disproportionately affected. Individuals aged 15–25 years account for a significant share of reported infections, and *Chlamydia trachomatis* prevalence is markedly higher in this age group than in older adults (5). These findings underscore the need for targeted STI prevention and education strategies for young Italians. Recent behavioral surveillance data reveal concerning trends. According to the Health Behavior in School-aged Children (HBSC) study, condom use among Italian adolescents has declined, with only 43% of young people aged 11–24 years reporting consistent condom use in 2023, while 30% report using neither condoms nor contraceptive pills at last intercourse. These national patterns underscore the persistent disconnect between STI awareness and protective behaviors among Italian youth (6).

Beyond their immediate physical health impact, STIs - particularly chronic infections - carry substantial psychological and sexual health consequences. Untreated or late-diagnosed infections may lead to pelvic inflammatory disease, infertility, ectopic pregnancy, and adverse pregnancy outcomes. They also increase the risk of infection-related cancers, such as cervical cancer from human papillomavirus (HPV) or hepatocellular carcinoma from hepatitis B and C viruses (1). Research has also documented significant associations between chronic STIs and symptoms of depression, anxiety, and impaired sexual functioning (7, 8). An Italian study found that individuals with chronic STIs experienced marked impairment in quality of life, mood disturbances, and sexual function compared to patients with chronic inflammatory bowel diseases (7). Similarly, international research using the WHOQOL-HIV/STI instrument has demonstrated that chronic STIs significantly impact multiple quality of life domains, with depression being particularly prevalent among affected individuals (8).

Given the high STI burden and frequent asymptomatic presentation among young adults, research has increasingly focused on STI-related knowledge, perceptions, and preventive behaviors among university students. This population represents a large and heterogeneous group of young adults at a critical life stage for sexual health. Despite their relatively high educational attainment, university students often demonstrate important knowledge gaps regarding STI transmission and prevention, as well as discrepancies between perceived and actual knowledge. A multi-centre Italian study of first-year university students found suboptimal awareness of STI prevention methods and inconsistent condom use, revealing critical gaps even among highly educated populations (9).

Despite the substantial evidence documenting STI-related knowledge gaps among university students, a critical question remains inadequately addressed: how do both objective and self-perceived knowledge vary across different behavioral risk profiles? Understanding this relationship is essential for designing targeted prevention strategies that match educational content to the specific needs and risk contexts of different student subgroups. International guidelines emphasize comprehensive, evidence-based sexual health education and targeted prevention strategies tailored to specific risk profiles as essential tools for reducing the STI burden among young people (1). Assessing both objective STI-related knowledge and self-perceived competence, alongside behavioral risk indicators, is crucial for identifying gaps and informing effective interventions.

University settings offer a strategic opportunity to explore these dimensions and support the design of prevention programs that promote safer sexual behaviors and reduce STI transmission among young adults.

The present study was conducted within the framework of the “Keep Calm and Stay Thoughtful and Informed (STI)” project. We examined STI-related knowledge and attitudes toward sexual health among university students in north-eastern Italy, with particular emphasis on differences across behavioral risk profiles. This stratified assessment was designed to inform the development of targeted, risk-adapted prevention strategies. Following established definitions, “knowledge” refers to awareness of facts and concepts related to STIs and sexual activity, while “attitude” represents “a relatively enduring organization of beliefs, feelings, and behavioral tendencies toward socially significant objects, groups, events or symbols” (10). This paper focuses specifically on knowledge-related findings; results regarding attitudes will be presented in a complementary paper.

To characterize STI knowledge among this population, we addressed four specific research questions:

1. To what extent are university students aware of specific STIs and able to identify risky sexual behaviors?
2. What are the primary sources from which students acquire STI-related information?
3. Which factors—including sociodemographic characteristics, educational background, and behavioral risk profile—influence students’ objective knowledge about STIs, including awareness of specific infections, understanding of risky behaviors, and knowledge of available protective measures?
4. Which factors influence students’ self-perceived knowledge about STI transmission and acquisition?

## 2 Materials and methods

### 2.1 Study design

This study employed a cross-sectional observational design using an ad-hoc survey.

### 2.2 Setting and population

All students enrolled in the University of Verona (Italy) during the academic year 2020–2021 were invited to participate. The university comprises eight faculties, offering degree programs with durations ranging from three to six years. No exclusion criteria were applied. In total, 22,982 students were invited to complete the survey.

### 2.3 Survey development

We designed an ad-hoc survey through the following steps:

1. Literature review: We reviewed existing studies on STI knowledge and attitudes to identify key content domains and priority topics. Based on this review, we developed original items ex novo to address the identified themes relevant to the Italian university context. No items were directly adopted or adapted from existing instruments; all questions were newly constructed to capture the specific research objectives of this study.
2. Expert evaluation: A panel of experts in health promotion and STI prevention evaluated the survey’s overall structure, item relevance, content gaps, and response format.
3. Survey refinement: We revised the draft based on expert feedback to achieve consensus. For STI-specific items, we selected conditions based on epidemiological data (higher prevalence or incidence among young adults) and expert input.
4. Pre-test and finalization: We tested the initial version with 23 university students from diverse academic backgrounds and age groups. Participants reviewed the survey and provided feedback via a 7-item pre-test questionnaire. This questionnaire assessed comprehensibility, acceptability, clarity, relevance, and completeness, with space for general suggestions. We refined the survey based on this input in collaboration with the research team.

The final questionnaire comprised 45 items across five main areas:

1. Sociodemographic and academic background (10 items).
2. Knowledge and awareness of STIs (8 items).
3. Information and education sources (6 items).
4. Attitudes toward STIs (16 items).
5. STI risk awareness related to aesthetic procedures (tattooing, piercing, cosmetic treatments) (5 items).

**Figure 1:**
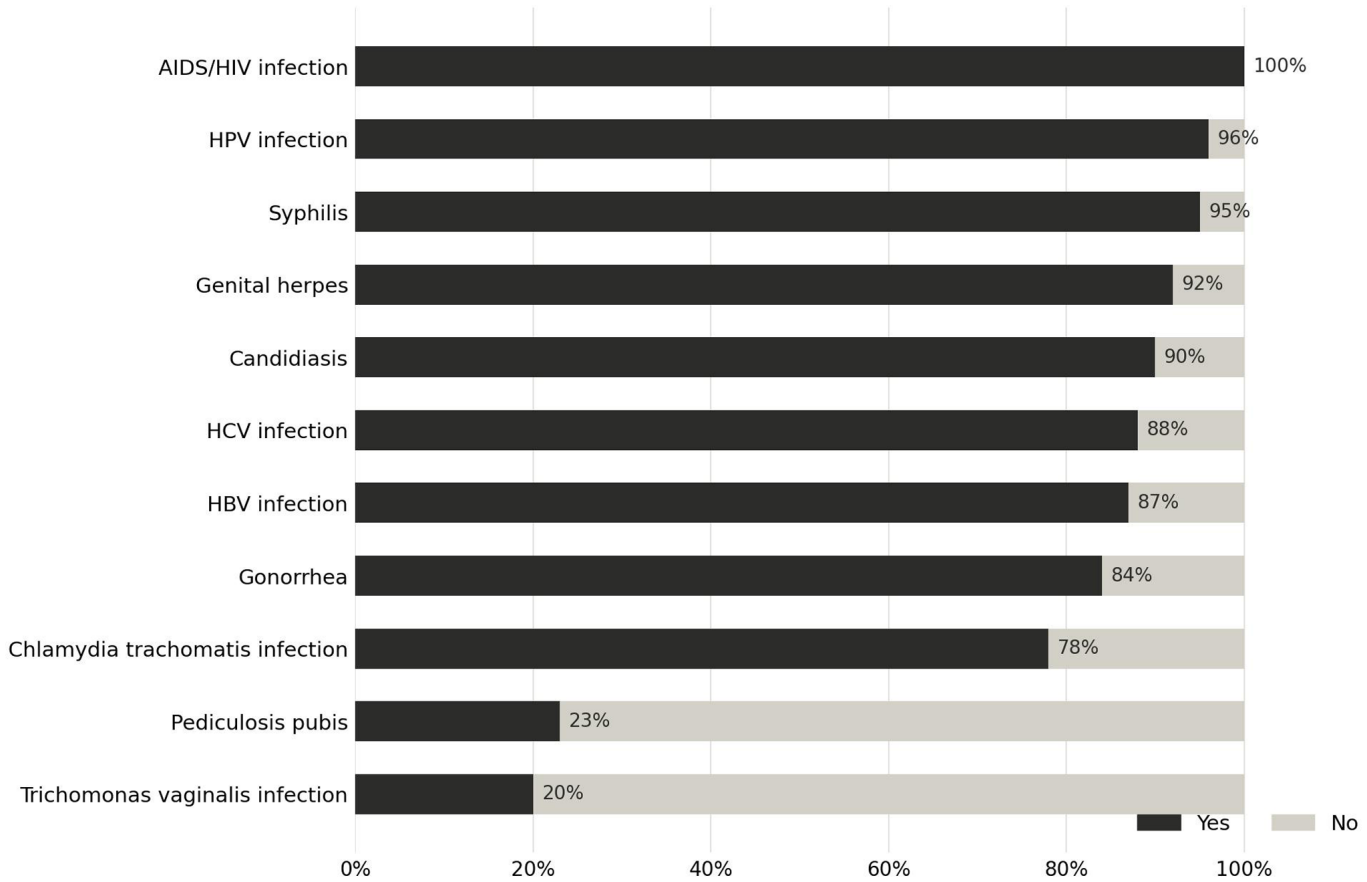
Proportion of study participants reporting awareness of specific STIs.

Specific questionnaire items are reported in the Results section within the corresponding tables and figures. Table 1 presents the exact wording of questions assessing knowledge of risky sexual practices and protective methods. Figure 1 lists the 11 specific STIs about which participants were asked. Self-perceived knowledge was assessed through direct questions asking students to rate their knowledge about “how to contract an STI” and “how to transmit an STI” on a five-point scale from “insufficient” to “excellent” (Table 2).

**Table 1:** Knowledge regarding sexual practices at risk of STI and ways to protect against infections.

Question		Answer, *n* (% of valid answers)		Valid answers
		Yes	No	
Do these sexual practices put you at risk of any STIs?	Vaginal intercourse	2,167 (99.5%)	10 (0.5%)	2,177
	Anal intercourse	2011 (93.5%)	141 (6.5%)	2,152
	Oral intercourse	1750 (81.8%)	389 (18.2%)	2,139
	Coitus interruptus	1935 (91.4%)	182 (8.6%)	2,117
	Mutual masturbation	692 (33.1%)	1,401 (66.9%)	2093
Do these methods provide protection against the risk of STIs?	Condom	2,165 (99.2%)	17 (0.8%)	2,182
	Morning-after pill/medicated plaster/contraceptive ring	139 (6.7%)	1947 (93.3%)	2086
	Spiral/contraceptive diaphragm	155 (7.5%)	1909 (92.5%)	2064
	Coitus interruptus	57 (2.7%)	2012 (97.3%)	2069
	Exclusively non-penetrative sexual practices	408 (19.6%)	1,677 (80.4%)	2085

Grey boxes indicate the correct answers.

**Table 2:** Self-assessed level of knowledge about how to contract or how to transmit an STI.

About how to contract an STI	About how to transmit an STI					
	Insufficient	Sufficient	Fair	Good	Excellent	Total
Insufficient	205 (86.1%)	26 (10.9%)	6 (2.5%)	1 (0.4%)	0	238
Sufficient	48 (10.8%)	333 (75%)	51 (11.5%)	10 (2.3%)	2 (0.5%)	444
Fair	11 (1.6%)	75 (10.6%)	535 (75.7%)	84 (11.9%)	2 (0.3%)	707
Good	0	2 (0.3%)	63 (9.5%)	577 (86.8%)	23 (3.5%)	665
Excellent	1 (0.8%)	0	1 (0.8%)	8 (6.7%)	109 (91.6%)	119
Total	265 (12.2%)	436 (20.1%)	656 (30.2%)	680 (31.3%)	136 (6.3%)	2,173 (100%)

### 2.4 Data collection

Data collection took place from December 2, 2020, to January 7, 2021. The survey was distributed digitally via the Lime Survey platform. Students were notified through personal emails sent to their university addresses and a push notification on the official University of Verona app (Univr App), accessible on smartphones, PDAs, and tablets. The communication included a brief project introduction and an invitation link directing students to a dedicated page with comprehensive survey details and a consent form.

Students who did not provide consent were automatically redirected to a page offering additional information and the option to complete the survey later. To encourage participation, international students received an English version of the questionnaire. A reminder was sent on December 22, 2020, via the same communication channels. Additionally, the study was promoted through digital platforms (Instagram, WhatsApp) with support from the students’ representatives.

All participants provided informed consent before taking part in the study. The survey was completed entirely anonymously, and data were analyzed in aggregate form to ensure participants’ confidentiality.

### 2.5 Risk stratification

We calculated each participant’s sexual activity profile by combining three variables: sexual activity status (“Have you ever had sexual intercourse?”), age at first sexual intercourse, and number of sexual partners in the past 12 months. Following established literature, we defined early sexual debut as onset before age 15 (11, 12). We defined multiple sexual partnerships as having sexual intercourse with more than one partner in the past 12 months (13).

We created a composite variable with four risk profiles (Table 3), which served as an indirect indicator of STI exposure risk. Participants who had never had sexual intercourse were classified as minimal risk. Those who started sexual activity at 18 years or older and had one or no partners in the past year were classified as low risk. We classified participants as medium risk if they either (a) started sexual activity at 18 years or older and had two partners in the past year, or (b) started between ages 15–17 and had two or fewer partners in the past year. Finally, we classified participants as high risk if they either started sexual activity before age 15 (regardless of partner number) or had three or more partners in the past year (regardless of age at first intercourse).

**Table 3:** Exposure risk to STIs as determined by the profile of sexual activity.

Variables			
Ever had sexual intercourse	Age at first sexual intercourse (years)	Sexual partners in the past 12 months (n)	Exposure
No	—	—	Minimal
Yes	≥18	≤1	Low
Yes	≥18	2	Medium
	15–17	≤2	
Yes	<15	Any value	High
	Any value	≥3	

### 2.6 Statistical analysis

We described participant characteristics using appropriate summary measures. We reported continuous variables as mean ± standard deviation or as median with interquartile range (IQR), and categorical variables as frequencies and percentages.

We performed four separate multivariable logistic regression models to evaluate factors associated with STI-related knowledge. Independent variables in all models included sex, age, sexual orientation, medical school attendance, and STI exposure risk (based on sexual activity profile). The dependent variables for each model were:

- Model 1: Awareness of all 11 STIs considered in the study (AIDS/HIV infection, HPV infection, syphilis, genital herpes, candidiasis, HCV infection, HBV infection, gonorrhoea, chlamydia trachomatis infection, pediculosis pubis, trichomonas vaginalis infection);
- Model 2: Correct knowledge about all STI-related questions (covering the infections listed above);
- Model 3: Self-reported knowledge rated as at least “sufficient” regarding how to contract an STI;
- Model 4: Self-reported knowledge rated as at least “sufficient” regarding how to transmit an STI.

Objective and self-perceived knowledge were analyzed as separate outcomes in independent models. This approach identified factors associated with each dimension but did not assess individual-level concordance between objective performance and self-rated competence. All statistical analyses were conducted using Stata version 18 (StataCorp LLC, College Station, TX, USA).

## 3 Results

### 3.1 Pre-test results and missing data assessment

The 23 students who participated in the pre-test had a median age of 25 years (mean = 23.7; SD = 3.3), with 60.9% identifying as female. They represented a wide range of different study areas. None of the participants found any items difficult to understand. Overall, 65% considered the items acceptable. The remaining participants noted that some questions could be potentially embarrassing but acknowledged their relevance to the study and felt reassured by the survey’s anonymity. No participant suggested adding or removing any items. Nearly all respondents (22 out of 23) found the topic either very interesting (*n* = 16) or quite interesting (*n* = 6). Clarity and comprehensiveness were rated as satisfactory. Only minor revisions were made following the pre-test.

### 3.2 Population

Of the 22,982 students invited, 2,258 completed the full questionnaire (response rate 9.8%). We excluded 71 participants (3.1%) who were over age 35 - as they represented non-traditional students whose risk profiles and educational contexts differ from the primary young adult population targeted by this study - or had unreported age, resulting in a final sample of 2,187 students. The median age was 21 years (mean 21.9; SD 2.8), and nearly all participants (2,099; 96%) were Italian nationals. Table 4 presents the full demographic characteristics of the study population.

**Table 4:** Characteristics of the study population.

Variable	*N* (%)
Age group (years)	
18–20	761 (34.8)
21–23	969 (44.3)
24–26	323 (14.8)
27–29	75 (3.4)
30–35	59 (2.7)
Gender	
Female	1,666 (76.2)
Male	505 (23.1)
Missing	16 (0.7)
Sexual orientation	
Heterosexual	1949 (89.1)
Homosexual	81 (3.7)
Other	148 (6.8)
Missing	9 (0.4)
Study area	
Health sciences	532 (24.3)
Nursing school	214 (9.8)
Medical school	178 (8.1)
Dental school	27 (1.2)
Health-allied professions	113 (5)
Foreign languages and literatures	346 (15.8)
Economics	321 (14.7)
Education, philosophy and social works	301 (13.8)
Science and engineering	254 (11.6)
Humanities, arts and communication	196 (9)
Law	119 (5.4)
Sports and exercise sciences	111 (5)
Missing	7 (0.3)
Year of study	
1st	777 (35.5)
2nd	467 (21.3)
3rd	471 (21.6)
4th	204 (9.3)
5th	173 (7.9)
6th or more	76 (3.5)
Missing	19 (0.9)

The majority of participants (1,737; 79.4%) reported having had sexual intercourse. Among these sexually active students, 1,720 provided information about their age at sexual debut: 114 (6.6%) initiated sexual activity before age 15, 803 (46.7%) between ages 15–17, and 803 (46.7%) at age 18 or older.

Regarding recent sexual activity, 1,732 sexually active participants reported their number of partners in the past year. Most (1,221; 70.5%) had one partner, while 220 (12.7%) had two partners, 152 (8.8%) had three or more, and 139 (8.0%) reported no sexual partner during that period.

We successfully classified 2,063 participants according to their sexual activity risk profile (124 participants, 5.7%, had incomplete data for risk classification). The distribution was: 346 (16.8%) at minimal risk, 668 (32.4%) at low risk, 802 (38.9%) at medium risk, and 247 (12.0%) at high risk.

Among the 1,737 sexually active participants, 1,727 reported their condom use frequency. Only 672 (38.9%) reported “always” using condoms, while 329 (19.1%) “never” used them. The remaining participants reported inconsistent use: 328 (19.0%) “almost always”, 224 (13.0%) “sometimes”, and 174 (10.1%) “almost never”.

We found significant associations between risk level and condom use frequency (*p* < 0.001). Participants at low risk reported the highest rate of consistent condom use (46.2% “always”). Similarly, condom use varied significantly with number of sexual partners (*p* < 0.001). Notably, participants with a single consistent partner or no partners in the previous year showed the highest proportions of consistent condom use (39.5 and 57.0%, respectively, reporting “always”) (Table 5).

**Table 5:** Condom use frequency by number of sexual partners.

Frequency of condom use during sexual intercourse	Number of sexual partners in the past 12 months				
	No partners	1	2	3 or more different partners	Total
Never	8 (5.8%)	285 (23.4%)	28 (12.7%)	8 (5.3%)	329 (19.0%)
Almost never	8 (5.8%)	134 (11.0%)	14 (6.4%)	18 (11.8%)	174 (10.1%)
Seldom	11 (8.0%)	130 (10.7%)	51 (23.2%)	32 (21.0%)	224 (13.0%)
Almost always	32 (23.4%)	188 (15.4%)	56 (25.5%)	52 (34.2%)	328 (19.0%)
Always	78 (57.0%)	481 (39.5%)	71 (32.3%)	42 (27.7%)	672 (38.9%)
Total	137 (100%)	1,218 (100%)	220 (100%)	152 (100%)	1727 (100%)

### 3.3 Knowledge and sources of information about STIs

When asked whether they had attended a university lecture addressing STIs, 321 students (15.5% of 2,073 valid responses) answered affirmatively. Of these, the vast majority (287; 89.4%) reported that this knowledge had influenced their survey responses.

All 2,187 participants had previously heard of STIs in general. However, awareness varied substantially depending on the specific infection (Figure 1). The median number of known STIs was 9 out of 11 (IQR: 8–9), but only 7.6% of participants reported awareness of all 11 STIs included in the survey.

*S*tudents’ knowledge regarding sexual practices associated with STI risks and protective methods is summarized in Table 1. Nearly all participants (99.5%) correctly identified vaginal intercourse as a risk, and most recognized anal intercourse (93.5%) and oral intercourse (81.8%) as risky practices. However, only one-third (33.1%) correctly identified mutual masturbation as a potential risk, and 8.6% incorrectly believed coitus interruptus eliminates STI risk.

Regarding protective measures, knowledge was similarly high for condoms (99.2% correct) but showed important gaps for other items. Several students incorrectly believed that hormonal contraceptives (6.7%), intrauterine devices or diaphragms (7.5%), and coitus interruptus (2.7%) provide STI protection. Approximately one-fifth (19.6%) correctly recognized that exclusively non-penetrative sexual practices reduce STI risk.

Overall, 816 participants (37.3%) answered nine out of ten questions correctly, while 504 (23.1%) answered all ten correctly.

When asked about specific aspects of proper condom use, 88.5% of participants (1,922 of 2,172) reported confidence in knowing when to put on a condom. However, this confidence decreased for knowing how to put it on (75.4%) and how to remove it (62.1%).

Table 2 shows how students rated their own knowledge about contracting and transmitting STIs. The distribution reveals generally coherent self-assessment: most students who rated their knowledge as insufficient for contracting STIs also rated it as insufficient for transmission (86.1%). Similarly, those rating their knowledge as excellent in one domain typically rated it as excellent in the other (91.6%).

**Figure 2:**
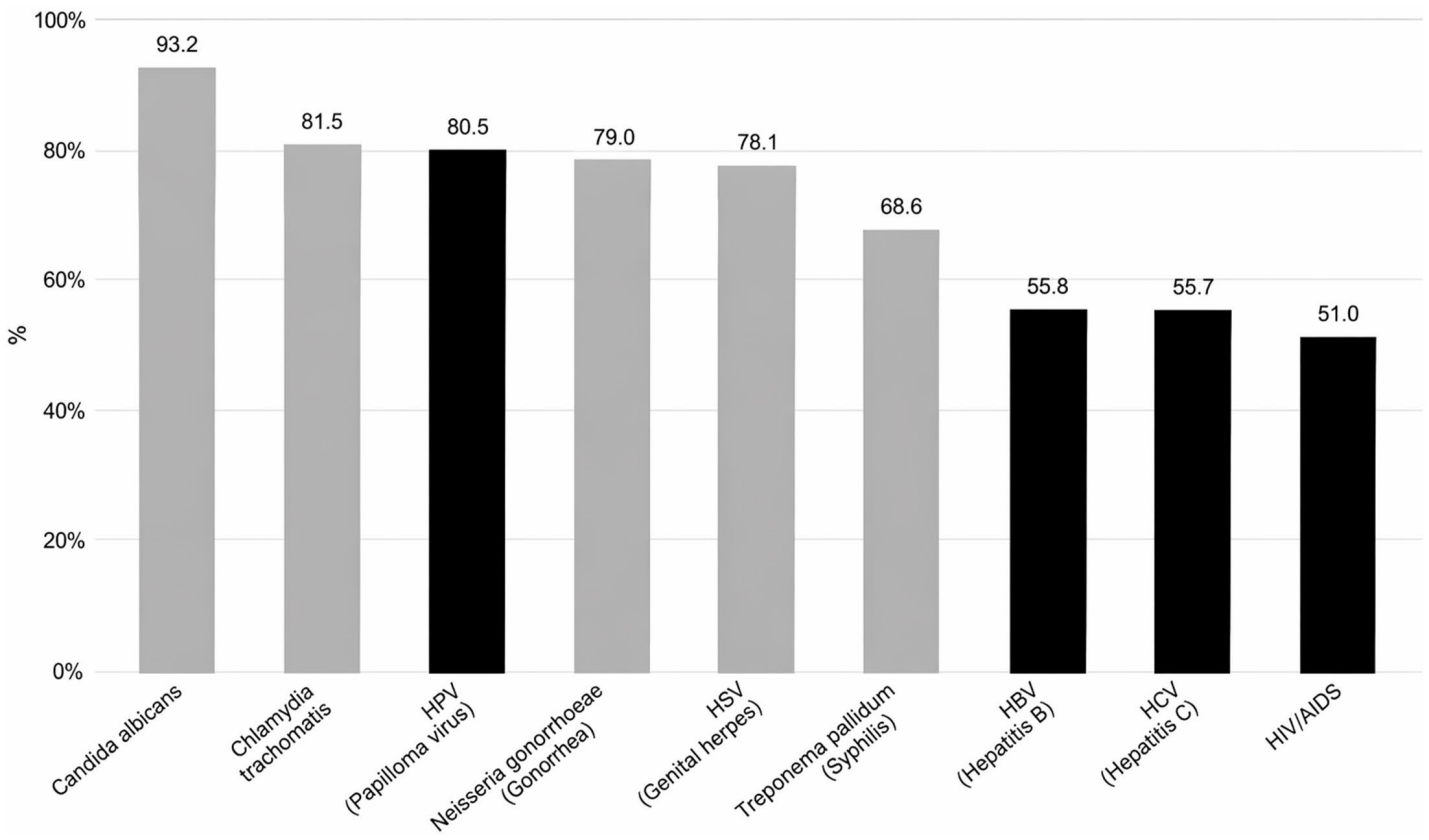
Proportion of respondents who correctly identified the relationship (or absence of) between specific STI and increased risk of cancer.

Questions about the relationship between specific STIs and cancer development showed the highest rate of missing responses (mean 14%). Figure 2 illustrates the proportion of participants who correctly identified whether STI for which an association with an increased cancer risk has been described in the literature. Infections with well-established or widely recognized oncological associations are highlighted in black. Knowledge varied considerably across different infections, with some associations better recognized than others.

Students reported obtaining general information about STIs from multiple sources. The internet was the most common source (89.2%; 1,920 of 2,153 valid responses), followed by university or school (57.9%), physicians or healthcare workers (54.2%), friends (39.7%), and family (27.4%).

When facing specific doubts or questions about STIs, students’ preferred sources differed. Nearly half (46.8%; 1,019 participants) indicated they would consult a specialist physician (gynaecologist, dermatologist, or other specialist), followed by the internet (35.8%; 780 participants), their general practitioner (11.5%; 251 participants), and friends or relatives (4.0%; 86 participants). Among the 41 participants (1.9%) who selected “other,” the majority (78.0%; 32 participants) indicated either healthcare professionals or scientifically reliable sources.

### 3.4 Factors associated with STI-related knowledge

The regression analyses revealed distinct patterns of association across four dimensions of STI knowledge, examined separately in Models 1–4. These included: awareness of all specific STIs (Model 1), correct knowledge of preventive methods (Model 2), self-perceived knowledge about contracting STIs (Model 3), and self-perceived knowledge about transmitting STIs (Model 4). The complete results are reported in Table 6, while the main findings are summarized below.

**Table 6:** Logistic regression models assessing factors associated with STI-related knowledge.

Variable	Model 1 Awareness of all the 11 STIs	Model 2 Correct prevention knowledge	Model 3 ≥Sufficient infection knowledge (self-assessment)	Model 4 ≥Sufficient transmission knowledge (self-assessment)
Age	1.22 (1.16–1.29) *p* < 0.001	1.08 (1.04–1.12) *p* < 0.001	1.02 (0.96–1.07) *p* = 0.530	1.03 (0.97–1.08) *p* = 0.330
Female	0.69 (0.45–1.04) *p* = 0.079	1.24 (0.95–1.61) *p* = 0.114	1.06 (0.76–1.48) *p* = 0.739	1.12 (0.81–1.54) *p* = 0.496
Homosexual	1.37 (0.63–2.96) *p* = 0.428	0.90 (0.51–1.61) *p* = 0.732	2.15 (0.76–6.08) *p* = 0.148	1.22 (0.54–2.75) *p* = 0.635
Other orientation	0.64 (0.26–1.57) *p* = 0.331	1.38 (0.92–2.05) *p* = 0.116	1.73 (0.91–3.28) *p* = 0.096	0.90 (0.54–1.50) *p* = 0.695
Medical school	14.52 (9.68–21.79) *p* < 0.001	1.97 (1.57–2.47) *p* < 0.001	2.65 (1.75–4.03) *p* < 0.001	2.45 (1.66–3.61) *p* < 0.001
Low risk	5.04 (2.09–12.12), p < 0.001	1.33 (0.94–1.89), *p* = 0.109	2.27 (1.53–3.39), p < 0.001	2.23 (1.53–3.27) p < 0.001
Medium risk	4.50 (1.87–10.81) *p* = 0.001	1.58 (1.13–2.21) *p* = 0.008	2.13 (1.47–3.08) *p* < 0.001	2.20 (1.54–3.15) p < 0.001
High risk	4.38 (1.64–11.70) *p* = 0.003	1.59 (1.05–2.42) *p* = 0.029	1.62 (1.00–2.65) *p* = 0.052	1.85 (1.15–2.98) *p* = 0.011

For each independent variable, the table shows the Odds Ratio, the 95% Confidence Interval and the *p*-value.

#### 3.4.1 Awareness of specific STIs (model 1)

Older participants were significantly more likely to know all 11 STIs (OR 1.22; 95% CI 1.16–1.29; *p* < 0.001). Medical school enrollment showed the strongest association with comprehensive STI awareness: medical students had over 14 times higher odds of knowing all STIs compared to non-medical students (OR 14.52; 95% CI 9.68–21.79; *p* < 0.001). Sexual activity risk profile was also strongly associated with STI awareness. Compared to those at minimal risk, participants at low risk (OR 5.04; *p* < 0.001), medium risk (OR 4.50; *p* = 0.001), and high risk (OR 4.38; *p* = 0.003) all showed significantly higher odds of comprehensive STI awareness. We found no significant associations with sex or sexual orientation.

#### 3.4.2 Knowledge of preventive methods (model 2)

Similar patterns emerged for correct knowledge of STI prevention. Older age (OR 1.08; *p* < 0.001) and medical school enrollment (OR 1.97; *p* < 0.001) were both significantly associated with correct prevention knowledge. Regarding risk profiles, participants at medium risk (OR 1.58; *p* = 0.008) and high risk (OR 1.59; *p* = 0.029) demonstrated significantly better prevention knowledge than those at minimal risk. The association for low-risk participants did not reach statistical significance (OR 1.33; *p* = 0.109). Sex and sexual orientation showed no significant associations with prevention knowledge.

#### 3.4.3 Self-perceived knowledge about contracting STIs (model 3)

Medical students were significantly more likely to rate their knowledge about contracting STIs as at least sufficient (OR 2.65; 95% CI 1.75–4.03; *p* < 0.001). All risk categories showed significantly higher self-perceived knowledge compared to minimal risk: low risk (OR 2.27; *p* < 0.001), medium risk (OR 2.13; *p* < 0.001), and high risk (OR 1.62; *p* = 0.052, marginally significant). Age, sex, and sexual orientation showed no significant associations with self-perceived knowledge about contracting STIs.

#### 3.4.4 Self-perceived knowledge about transmitting STIs (model 4)

The pattern for self-perceived transmission knowledge closely mirrored that of contraction knowledge. Medical students again showed significantly higher odds of rating their knowledge as at least sufficient (OR 2.45; 95% CI 1.66–3.61; *p* < 0.001). Participants at low risk (OR 2.23; *p* < 0.001), medium risk (OR 2.20; *p* < 0.001), and high risk (OR 1.85; *p* = 0.011) all reported significantly higher self-perceived transmission knowledge compared to those at minimal risk. As with Model 3, age, sex, and sexual orientation were not significantly associated with the outcome.

## 4 Discussion

This study provides a comprehensive assessment of STI-related knowledge among university students in north-eastern Italy, examining both objective knowledge and self-perceived competence alongside behavioral risk indicators. Our findings reveal substantial variability in STI knowledge across different student populations and highlight the complex interplay between education, sexual behavior, and health literacy.

Three key patterns emerged from our analysis. First, medical students demonstrated markedly superior knowledge compared to their non-medical peers across all measured dimensions. Second, sexually active participants - particularly those at higher risk - showed greater STI awareness than those with minimal sexual experience. Third, self-perceived knowledge aligned more closely with educational background and sexual experience than with demographic characteristics such as age, gender, or sexual orientation.

The striking difference between medical and non-medical students represents one of our most robust findings. Medical students showed 14-fold higher odds of knowing all 11 STIs and approximately 2.5-fold higher odds of rating their own knowledge as sufficient. This finding aligns with recent European studies documenting similar patterns across multiple contexts. A Serbian study of 1,273 university students found that 29.1% of medical students could name four of five common STIs compared to only 13.4% of non-medical students (14). Similarly, research from Greece reported that medical students demonstrated significantly better knowledge than nursing students across multiple STI domains, with medical students more frequently able to list widely known STIs (15). However, this educational advantage raises important public health concerns. If comprehensive STI knowledge remains largely confined to health sciences students, the broader university population - representing diverse future professionals and community leaders - may lack adequate sexual health literacy. This gap is particularly troubling given that non-medical students constitute the vast majority of the university population and yet face similar STI risks. These findings underscore the need for university-wide sexual health education programs that extend beyond health sciences curricula.

Our finding that participants at higher sexual risk demonstrated greater STI knowledge presents an intriguing paradox. Those classified as low-, medium-, or high-risk showed significantly better knowledge and awareness of specific STIs and prevention methods compared to those at minimal risk. Several explanations merit consideration.

First, sexually active individuals may actively seek STI information due to perceived personal relevance - a phenomenon consistent with health behavior models emphasizing the role of perceived susceptibility in motivating information-seeking. Second, sexual experience itself may serve as an informal learning opportunity through partner discussions, healthcare encounters, or peer conversations. Third, individuals who engage in higher-risk behaviors may possess pre-existing characteristics (such as greater openness to sexual health topics or lower stigma) that facilitate both knowledge acquisition and sexual activity.

However, this association should not be interpreted as evidence that knowledge prevents risky behavior. Our data show that despite higher knowledge levels, participants at greater risk did not demonstrate proportionally safer practices. For instance, consistent condom use was actually lowest among those with multiple partners - precisely the group at highest STI risk. This disconnection between knowledge and behavior has been documented in sexual health research and highlights the limitations of information-based interventions alone (16–18). Recent studies corroborate this finding. Research demonstrates that while STI knowledge correlates with safer intentions, the translation to actual protective behaviors depends on multiple factors including self-efficacy, partner communication skills, access to prevention resources, and social norms (19, 20). Public health interventions must therefore address these behavioral determinants alongside knowledge gaps.

Older participants showed significantly better objective knowledge about STIs and prevention methods, but this age effect disappeared for self-perceived knowledge. This pattern suggests that age-related knowledge gains may reflect cumulative exposure to sexual health information and life experience rather than fundamental differences in confidence or self-assessment. The age gradient in objective knowledge aligns with existing literature documenting progressive knowledge accumulation through young adulthood. However, the absence of age effects on self-perceived knowledge indicates that older students do not necessarily feel more competent despite knowing more - a potential indicator of increased awareness of knowledge gaps as individuals gain experience. This finding has practical implications for prevention programs. While younger students may benefit from foundational education, older students might profit more from advanced topics addressing their evolving needs and questions.

Notably, when controlling for age, medical school enrollment, and behavioral risk profile, neither sex nor sexual orientation showed significant associations with STI knowledge in any of our models. This contrasts with some previous studies reporting gender differences in sexual health literacy, though findings have been inconsistent across contexts. The absence of gender effects in our adjusted models suggests that apparent gender differences in STI knowledge may largely reflect differences in educational pathways (more women in health sciences) or sexual behavior patterns rather than gender per se. This finding is encouraging from an equity perspective, suggesting that within university settings, knowledge acquisition opportunities may be relatively gender-neutral. Similarly, the lack of significant associations with sexual orientation challenges assumptions about differential STI awareness among sexual minority groups. While some research has suggested that sexual minority individuals demonstrate heightened awareness of certain STIs (particularly HIV), our findings indicate that, within this university context, sexual orientation does not independently predict comprehensive STI knowledge.

While we could not directly compare objective and self-perceived knowledge at the individual level, the different patterns of associations provide insights. Medical students and sexually active participants consistently reported higher self-perceived competence - groups that also demonstrated superior objective knowledge. However, the absence of age effects on self-perceived knowledge despite clear age-related improvements in objective knowledge suggests potential calibration issues. This pattern raises questions about metacognitive accuracy in sexual health. Do younger students with less knowledge overestimate their competence, or do older students with more knowledge appropriately recognize remaining gaps? Research on health literacy suggests that individuals with limited knowledge often struggle to accurately assess their competence - a phenomenon known as the Dunning-Kruger effect (21, 22). Studies have demonstrated that individuals with low health literacy report equal or greater confidence in their health knowledge than those with higher health literacy, and yet exhibit more problematic health behaviors (21). This metacognitive bias has been documented across various health domains, suggesting that those who most need health education may be least aware of their knowledge deficits (23, 24). Understanding concordance between perceived and actual knowledge has important implications for intervention design. Individuals who underestimate their knowledge gaps may not seek additional information, while those who accurately recognize deficiencies are more likely to engage with educational resources.

Our finding that only 38.9% of sexually active participants reported consistent condom use is concerning, particularly given relatively high awareness of STI transmission routes. Even more troubling, participants with multiple partners - those at highest risk - showed lower rates of consistent condom use (27.7% reporting “always”) compared to those with single partners (39.5%). This inverse relationship between risk and protection has been documented in other European studies and represents a critical public health challenge (25). Possible explanations include risk compensation [individuals with multiple partners may use other contraceptive methods prioritizing pregnancy prevention over STI protection (26)], partner-specific risk assessment, [i.e., perceiving some partners as “safe” (27)], reduced negotiation power in casual relationships (28), or alcohol and substance use impairing judgment. Recent research emphasizes that improving condom use requires interventions addressing not just knowledge but also skills in partner communication, confidence in negotiation, access to condoms, and social norms around condom use (16, 17). Universities represent ideal settings for such comprehensive approaches, as systematic reviews demonstrate that school-based sexuality education interventions can effectively improve sexual health behaviors when they incorporate behavior change techniques alongside information provision (29, 30).

The disconnection between STI knowledge and protective behaviors requires consideration of underlying mechanisms. The Health Belief Model suggests that knowledge alone is insufficient; students may possess factual awareness while engaging in optimistic bias (“it won’t happen to me”) or perceiving specific partners as low-risk. The Theory of Planned Behavior highlights additional barriers: students may lack confidence in negotiating condom use, fear relationship conflict, or perceive peer norms as accepting unprotected sex. Situational factors frequently disrupt knowledge-based intentions. Our finding that consistent condom use was lowest among those with multiple partners suggests complex partner-specific risk assessments override general knowledge. Additionally, students may engage in risk compensation when other contraceptive methods address pregnancy concerns. Specifically, individuals with multiple partners and higher STI knowledge may perceive alternative prevention strategies—such as regular STI testing, prompt treatment or pre-exposure prophylaxis (PrEP) where available—as adequate risk management, reducing perceived need for consistent condom use. Paradoxically, greater knowledge of STI signs, treatments, and curability may diminish perceived severity and urgency of prevention, particularly for bacterial STIs that are readily treatable. This knowledge-mediated risk compensation suggests that education emphasizing treatability without equally stressing prevention may inadvertently reduce protective behaviors among more informed individuals. Effective interventions must therefore extend beyond information provision to address self-efficacy in partner communication, normative perceptions, and practical negotiation skills within real-world sexual contexts.

The predominance of the internet as an information source (89.2% of participants) while reassuring in terms of access, raises questions about information quality. While many students indicated they would consult healthcare professionals for specific concerns (46.8%), the internet served as the primary source of general information. This pattern reflects broader trends in health information-seeking among young adults, who increasingly turn to online sources for health queries (18). However, research examining online STI resources reveals substantial quality concerns. A comprehensive review of STI-related mobile applications found marked variation in content, quality, and accuracy, with nearly one-third containing potentially harmful information (31). Similarly, studies of online sexual health information have documented inconsistent reliability and frequent gaps in comprehensive coverage (32). The challenge for public health is not to redirect students away from online sources but to improve the quality and accessibility of reliable digital resources while enhancing students’ ability to critically evaluate information quality. Evidence suggests that interventions promoting positive language about sexuality and addressing sexual pleasure alongside safety are more effective in engaging young people (33).

Our findings suggest several directions for university sexual health programs: interventions could extend beyond health sciences students to reach the broader university population; programs could address not only knowledge gaps but also the behavioral, social, and structural factors influencing protective behaviors; and, age-appropriate programming could recognize that students’ needs evolve throughout their university careers. Effective interventions might include mandatory sexual health modules integrated into first-year orientation programs, peer education initiatives leveraging the influence of student networks, accessible confidential testing and counseling services, and partnerships with student organizations to reduce stigma and normalize sexual health discussions. Digital platforms, given their widespread use, represent promising channels for delivering tailored, evidence-based content (34, 35). Recent systematic reviews and meta-analyses demonstrate that comprehensive sexuality education programs, particularly those incorporating multiple delivery methods including digital interventions, can effectively improve knowledge, attitudes, and protective behaviors among university-age populations (36, 37). Recent evidence supports comprehensive approaches combining education, skills training, and environmental modifications (38, 39). Universities implementing such multi-level interventions have reported improvements in both knowledge and protective behaviors, though sustained effects require ongoing reinforcement (40). Evidence from diverse contexts suggests that successful programs share common characteristics: they are theory-based, address multiple behavioral determinants beyond knowledge alone, use interactive methods that engage students actively, and are culturally appropriate to the target population (41).

### 4.1 Strengths and limitations

This study’s main strengths include its large sample size, assessment of multiple knowledge dimensions, and simultaneous consideration of objective and self-perceived knowledge alongside behavioral indicators. The inclusion of participants from diverse academic backgrounds provides insights applicable across university settings, while the multivariable analytical approach permitted assessment of independent associations.

However, several limitations warrant acknowledgment. Our approach examined objective and self-perceived knowledge as separate outcomes rather than conducting paired individual-level concordance analyses, preventing direct assessment of whether students accurately calibrate their self-assessments. Additionally, knowledge assessments did not capture all aspects of sexual health literacy, such as practical skills in accessing care or partner communication.

The modest response rate raises concerns about potential selection bias, as students with greater interest in sexual health may have been more likely to participate. However, our sample size provided adequate statistical power, demographic characteristics broadly align with official university statistics, and our findings align with patterns documented in comparable studies with higher response rates. Nevertheless, readers should interpret prevalence estimates with appropriate caution.

The predominance of female participants reflects the actual demographic composition of the University of Verona (more than 60% female enrollment, with higher proportions in certain faculties). While this may limit generalizability to male-dominant settings, our multivariable analyses revealed that sex was not a significant independent predictor of STI knowledge after adjusting for educational pathway and behavior, suggesting our key findings may be generalizable across genders. Similarly, the high proportion of Italian nationals (96%) limits generalizability to more internationally diverse university settings where cultural backgrounds and prior education may differ.

Self-reported data may be subject to social desirability and recall biases, particularly for sensitive behaviors. The timing of data collection (December 2020–January 2021, during COVID-19) may have influenced behaviors and information-seeking patterns.

Despite these limitations, our findings provide valuable insights into STI knowledge patterns and identify educational disparities that warrant attention in public health programming. The convergence of our results with international studies strengthens confidence in our key findings regarding the medical versus non-medical student knowledge gap and the disconnection between knowledge and protective behaviors.

## 5 Conclusion

This study documents substantial variation in STI-related knowledge among university students. Medical students and sexually active individuals showed higher knowledge levels and greater confidence across multiple dimensions, though these associations do not establish causality. Knowledge gaps were observed even among highly educated populations, and disconnects existed between knowledge and protective behaviors.

These findings highlight potential opportunities for comprehensive, university-wide sexual health education extending beyond traditional health sciences curricula. Future interventions may benefit from multi-level approaches addressing not only knowledge deficits but also behavioral skills, social norms, and structural barriers. Longitudinal research is needed to establish temporal relationships and explore mechanisms linking knowledge, risk perception, and behavior. Given the persistent nature of STI transmission among young adults and universities’ unique access to this population, evidence-based sexual health programming represents an important public health priority. Universities are well-positioned to support students in developing the knowledge, skills, and resources necessary to protect their sexual health.

## Data Availability

The raw data supporting the conclusions of this article will be made available by the authors, without undue reservation.
